# Is neighborhood socioeconomic status associated with health behavior in Berlin? Cross-sectional data of the German National Cohort (NAKO)

**DOI:** 10.1186/s12889-026-26734-5

**Published:** 2026-02-23

**Authors:** Lilian Krist, Kathrin Wolf, Matthias B. Schulze, Tobias Pischon, Florian Herbolsheimer, Karen Steindorf, Thomas Keil

**Affiliations:** 1 Institute of Social Medicine, Epidemiology and Health Economics, Charité - Universitätsmedizin Berlin, corporate member of Freie Universität Berlin and Humboldt-Universität Zu Berlin, Luisenstr. 57, Berlin, 10117 Germany; 2 Institute of Epidemiology, Helmholtz Zentrum München GmbH, German Research Center for Environmental Health, Ingolstädter Landstraße 1, Oberschleißheim, Neuherberg, 85764 Germany; 3 Department of Molecular Epidemiology, German Institute of Human Nutrition Potsdam-Rehbruecke, Arthur-Scheunert-Allee 114-116, Nuthetal, 14558 Germany; 4 Institute of Nutritional Science, University of Potsdam, Arthur-Scheunert-Allee 114-116, Nuthetal, 14558 Germany; 5 Charité - Universitätsmedizin Berlin, corporate member of Freie Universität Berlin and Humboldt-Universität Zu Berlin, Charitéplatz 1, Berlin, 10117 Germany; 6 Molecular Epidemiology Research Group, Max-Delbrueck-Center for Molecular Medicine in the Helmholtz Association (MDC), Robert-Rössle-Straße 10, Berlin, 13125 Germany; 7 Division of Physical Activity, Cancer Prevention and Survivorship, German Cancer Research Center (DKFZ), Im Neuenheimer Feld 280, Heidelberg, 69120 Germany; 8 Institute of Clinical Epidemiology and Biometry, University of Würzburg, Sanderring 2, Würzburg, 97070 Germany; 9 Institute for Health Resort Medicine and Health Promotion, Bavarian Health and Food Safety Authority, Prinzregentenstraße 6, Bad Kissingen, 97688 Germany

**Keywords:** Neighborhood, Socioeconomic status, Healthy lifestyle index, Germany, NAKO, Accelerometry

## Abstract

**Background:**

Neighborhood socioeconomic status (nSES) can complement individual SES to better assess health-behavior inequalities. The aim of this study was to investigate the relationship between the nSES of defined areas in Berlin with healthy lifestyle.

**Methods:**

This cross-sectional analysis used baseline data from the three Berlin study centers of the German National Cohort (NAKO). We assessed body mass index (BMI), smoking, alcohol consumption, and objectively measured physical activity and combined them to a healthy lifestyle index (HLI; range:0–12 points; 12 = best score). To assess nSES, the Social Index from Berlin’s Social Structure Atlas (1 = best; 7 = worst) was assigned to the participants’ residential locations. We used multivariable regression analyses to examine the association between nSES and the HLI (mean difference with 95% confidence interval, CI) as well as the four individual lifestyle factors (odds ratios (OR) with 95% CI). In sensitivity analyses, nSES was modelled using all seven Social Index categories and as a dichotomy (categories 1–4 vs. 5–7).

**Results:**

Of 204,801 NAKO participants, 31,075 were recruited in Berlin, of those 11,922 with complete accelerometry data were included (mean ± SD age 50.6 ± 12.9 years; 52.8% women). The mean HLI was 8.3 ± 2.0 points. Worsening of nSES by one point was associated with a 0.08-point lower HLI (-0.08 (95%-CI -0.10; -0.06)), with a reduced odds of normal weight (0.95; 0.93–0.97) and being a never-smoker (0.96; 0.94–0.98), while it was neither associated with alcohol consumption (1.01; 0.99–1.04)) nor physical activity (0.99; 0.97–1.02)). Sensitivity analyses suggested that differences were mainly driven by a contrast between categories 1–4 and the more disadvantaged categories 5–7. However, the overall pattern of results did not change.

**Conclusions:**

Our analyses suggest a rather small association between Berlin’s nSES and HLI, and slightly stronger associations with BMI and smoking. Future studies using longitudinal data and more neighbourhood measures are needed to better disentangle contextual influences from residential selection and to inform targeted prevention strategies.

**Supplementary Information:**

The online version contains supplementary material available at 10.1186/s12889-026-26734-5.

## Introduction

There is a positive association between the individual socioeconomic status (SES), represented by educational achievement, employment, or income, and health [1, 2]. Reasons for that have been investigated in the past showing that education is correlated with different domains such as the work and economic situation, social and psychological resources, and healthy lifestyle which can all influence health [3]. While the evidence on the unfavorable association of low SES with outcomes such as smoking and high body weight is relatively consistent, findings on the relationship between SES and alcohol consumption or physical activity (PA) remain heterogeneous [4, 5]. High SES can also have unfavorable effects, e.g. being associated with less sleep and higher sedentary time [6].

When investigating SES as exposure or confounding variable, it seems to be important not to focus on just one measure of SES, but to consider individual as well as neighborhood SES (nSES) in order to avoid residual confounding [7]. A recent study reported a widening life-expectancy gap between the least- and most-deprived areas in Germany [8]. Understanding the underlying reasons of these disparities is therefore urgent. NSES is operationalised in different ways including most often aggregates of income, labour market participation, occupational status, welfare support, and educational attainment [9]. Beyond individual SES, nSES has been shown to influence health and health behavior through limited resources, increased stress, and negative perceptions of the environment [10, 11]. Moreover, low nSES appears to impose a comparatively smaller burden on individuals with high individual SES than on those with low individual SES, as the latter are more reliant on neighborhood support structures [12]. A recent meta-analyses confirmed this by showing that neighbourhood green space, built physical activity facilities, and walking and cycling infrastructure were positively associated with PA only in persons with low SES [13]. Ribeiro et al. demonstrated that nSES significantly predicts mortality risk, with this association being markedly stronger among individuals of lower individual SES [14]. In another analysis, only the economic domain of nSES was significantly associated with increased mortality risk [15]. Regarding health behavior, a systematic review found that physical activity and smoking were associated with nSES, while alcohol consumption and nutrition were not [16].

Several German studies reported associations of nSES with diabetes prevalence, life satisfaction as well as quality of life [17–19]. In 2017, the Robert Koch-Institute has developed the German Index of Socioeconomic Deprivation (GISD) to be used by researchers and health authorities [20]. It shows regional socioeconomic differences and is intended to help understand and reduce social inequalities in Germany. A revised version is available as from 2022 [21]. German studies using the GISD report differences in lifestyle behavior, risk of diabetes, and life expectancy with premature cancer and overall mortality in persons with low nSES [8, 22–24]. No differences could be shown for mental health in older persons [25]. At the moment, GISD is, however, only available at the municipal level and therefore, does not allow the spatial disparities within a city which can be largely heterogenous. Berlin is the German capital and with 891 square kilometers the largest municipality in Germany [26], and with around 3.7 million inhabitants it is the most populous city in Germany and in the European Union [27, 28]. The Berlin Senate Department for Health and Social Affairs has implemented the "Atlas of social structure (Sozialstrukturatlas)” that has shown differences in income, employment status, but also in life expectancy of more than 2 years between different districts of Berlin [29]. However, no detailed individual data on lifestyle factors or health status is available for the inhabitants of Berlin in this report.

The main aim of this study was therefore to investigate the association of nSES with a healthy lifestyle using a combined index while adjusting for the individual socioeconomic status in a sample of persons living in different neighborhoods of Berlin. Additionally, we aimed to map objectively measured health behaviors within Berlin city districts for the first time combining population-based NAKO data with data from the Atlas of social structure of Berlin.

## Methods

### Study design and sample

We performed a cross-sectional analysis using the Berlin subsample of the German National Cohort Study (NAKO). NAKO is a nation-wide population-based prospective, and ongoing cohort study with more than 204,000 participants aged 20–69 years at the time of baseline recruitment (2014–2019) [30]. The overall aim of the study is to investigate risk factors, and risk prediction of a broad range of chronic diseases and to provide data bases for the development of prevention measures. The study is conducted in urban and rural regions across Germany represented by 18 study centers, among those three study centers in Berlin: Berlin-North (recruiting participants living in the northern districts of Berlin and in some regions of the Land Brandenburg), Berlin-Center (recruiting in the central disctricts of Berlin), and Berlin-South/Brandenburg (recruiting in the southern districts of Berlin and some regions of the Land Brandenburg). The study design intended to recruit 10% of participants in each 10-year group between 20 and 39 years of age and 26.7% in each 10-year group between 40 and 69 years of age with 50% men and women in all of these groups. Participants were randomly selected from population registries and invited to the study centers. The overall response was 15.6%, (Berlin-North: 11.2%, Berlin-Center: 13.7%, Berlin South/Brandenburg: 7.6%) [31]. The baseline assessment included standardized interviews, self-completed questionnaires, in-depth physical and medical examinations with clinical biomarker measurements, and biosample collection. In addition, hip-worn accelerometers were handed out to a random sample of 50% of all participants [32]. The study was approved by the ethical review committee of the Charité-Universitätsmedizin Berlin. All participants gave their written informed consent. For the present analysis, only data of participants living in Berlin were included.

### Outcomes

#### Lifestyle variables

For calculating the BMI, trained study staff measured body height to the nearest 0.1 cm and body weight to the nearest 0.1 kg using a calibrated integrated measurement station (SECA model 764, Seca®, Hamburg, Germany). BMI was then calculated as weight over height squared in kg/m^2^ and categorized into underweight (BMI < 18.5 kg/m^2^), normal weight (BMI 18.5–24.9 kg/m^2^), overweight (BMI 25.0–29.9 kg/m^2^), obesity class 1 (BMI > 30.0–34.9 kg/m^2^), and obesity class 2 or higher (BMI ≥ 35 kg/m^2^). Due to the very low percentage of participants with underweight (1.4%), underweight and normal weight were combined to normal weight (BMI under 25 kg/m^2^).

Smoking status was assessed via self-report and categorized into never-smokers, former smokers, light regular smokers (< 15 cigarettes per day), and heavy regular smokers (≥ 15 cigarettes per day).

Alcohol consumption was assessed via self-report as well and categorized according to the short form of the Alcohol Use Disorders Identification Test (AUDIT-C) definition into low risk (AUDIT-C-Score 0–2 or 0–3 for women and men, respectively), moderate risk (3–5 or 4–5 for women and men, respectively), high risk (6–7), and severe risk (8–12) drinkers [33].

Physical activity was measured by hip-worn accelerometers (GT3X/+, Fa. ActiGraph, Pensacola, FL, USA). Participants received the accelerometer at the study center where it was placed at the hip on the side of the dominant hand attached with an elastic belt. They were advised to wear it for seven consecutive days and nights, but were allowed to take it off for showering or swimming. Tri-axial raw accelerometry data were collected at a sampling rate of 100 Hz and processed using the open-source R package GGIR version 2.10–3. Data processing is described in more detail elsewhere [34]. Valid wear time was defined as at least 16 h per day and at least two valid weekdays and one valid week-end day. Data from participants who did not meet these criteria were excluded. According to the cut-offs proposed by Weber et al., moderate-to-vigorous physical activity (MVPA) minutes per day were calculated using the mean average (epoch length 60 s, no bouts, Mean Amplitude Deviation (MAD) threshold = 90 mg) and then categorized into quartiles.

#### Healthy Lifestyle Index

**Fig. 1:**
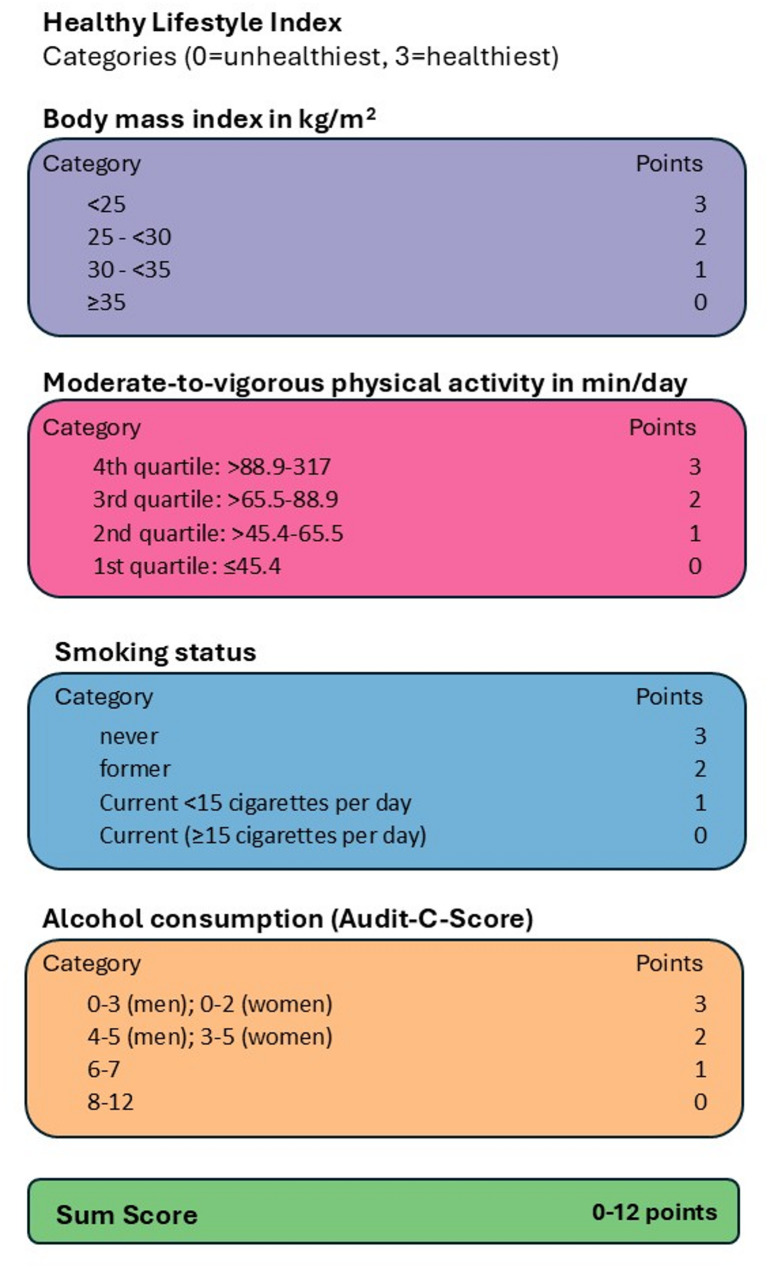
Calculation of the healthy lifestyle index

Healthy lifestyle index (HLI) covered the factors body mass index (BMI), moderate-to-vigorous physical activity, smoking status, and alcohol consumption. Each factor comprised a score ranging from 0 to 3, with 0 being the least and 3 being the most healthy behavior. The HLI was calculated as sum of all 4 scores, ranging from 0 to 12. For the calculation of the score see Fig. 1 and Supplementary Table 1.

For nSES, we used the Social Index I, which is a composite indicator for social deprivation provided by the “Sozialstrukturatlas Berlin 2013” [29]. Berlin is divided into 12 districts. The atlas further divides Berlin into 60 so-called prognosis areas, 138 district regions, and 447 planning areas. For our analyses, we used the least granulated classification, namely the prognosis area. Due to data protection reasons, only prognosis areas with at least 20 participants were included resulting in 53 out of 60 analyzable prognosis areas. For each of these areas, social indicators are created to characterize the areas in terms of socio-economic parameters such as unemployment rate or prevalence of state transfer payments. Additionally, in contrast to the GISD, the social structure atlas includes smoking behavior and some key health indicators such as premature and preventable mortality. Index values range from 1 to 7 with lower values reflecting more favorable socio-economic conditions. The Social Index was assigned to the NAKO data based on the geocoded residential addresses of the participants. More details on the geocoding and linkage with health data can be found elsewhere [35]. For the aggregation of individual data to the prognosis area level, we combined all participants whose residential addresses fell within the same prognosis area. For BMI and physical activity, we calculated the mean value of participants per prognosis area,for regular smoking and risky alcohol consumption, we calculated the proportion.

### Covariates

As covariates we included sex (male/female), age, marital status (living with partner, not living with partner, no partner), education in years and categorized into “high”, “middle” and “low” according to ISCED-97 guidelines, net household income in Euro, employment status (employed, not employed, economically inactive (retired or other reasons)), and Turkish descent (own Turkish background or at least one parent migrated from Turkey to Germany after 1949) (yes/no) [36], since this is the largest migrant group in Berlin [37]. Potential environmental pathways are illustrated in a conceptual framework (Supplementary Fig. 2),however, related variables were not available and therefore could not be adjusted for.

### Statistics

We considered our statistical approach to be explorative rather than strictly hypothesis-testing. Participants’ sociodemographic and lifestyle characteristics were summarized using means and standard deviations (SD) for continuous variables and absolute and relative frequencies for categorical data. Missing data were not imputed. As part of the descriptive analyses, we compiled maps to visualize the Social Index as well as the HLI and the individual lifestyle factors aggregated over the prognosis areas of Berlin. These maps were intended as exploratory visualisations to describe the spatial distribution of nSES and lifestyle factors and to generate hypotheses, not to provide formal statistical inference. The main exposure was neighbourhood socioeconomic status (nSES), operationalised by Berlin’s Social Index (Social Index I; higher values indicate lower nSES).

Multivariable regression analyses (adjusted for age, sex, education level, employment, and Turkish descent) were used to examine the association between nSES and the i) HLI (presented as mean difference with 95% confidence interval, CI), and ii) the four individual lifestyle factors (presented as odds ratios (OR) with 95% CI). For analyses of the individual lifestyle factors, outcomes were dichotomised into beneficial vs. not beneficial: BMI: < 25 kg/m^2^ vs. ≥ 25 kg/m^2^, PA: upper median vs. lower median, smoking: never vs. ever, alcohol consumption: not risky (≤ 4 (men), ≤ 3 (women)) vs. risky (> 4 (men), > 3 (women)).

To investigate a possible effect modification of individual SES, we fit generalized linear models with a binomial–logit link including main effects for the exposure (nSES) and the putative moderator (individual SES) and their interaction, adjusted for all covariates of the regression model; the scale parameter was fixed at 1, Type-III likelihood-ratio tests provided inference, and stratum-specific contrasts were obtained from estimated marginal means on the logit scale and reported as odds ratios. In the primary analyses, nSES was entered as an ordinal predictor (linear trend across the Social Index categories). In reponse to peer-review and to assess potential departures from linearity, we additionally re-specified nSES as a categorical variable with all seven Social Index categories (indicator coding; category 1 as reference). Based on the pattern observed in this analysis, we further conducted a sensitivity analysis collapsing nSES into two groups (categories 1–4 vs. 5–7) to quantify the contrast between higher/middle versus more disadvantaged prognosis areas; because this regrouping was data-driven, it was treated as a sensitivity analysis. Finally, to explore potential effect modification, we conducted sex-stratified analyses and tested for nSES × sex interaction in pooled models.

## Results

### Characteristics of the study sample

**Fig. 2:**
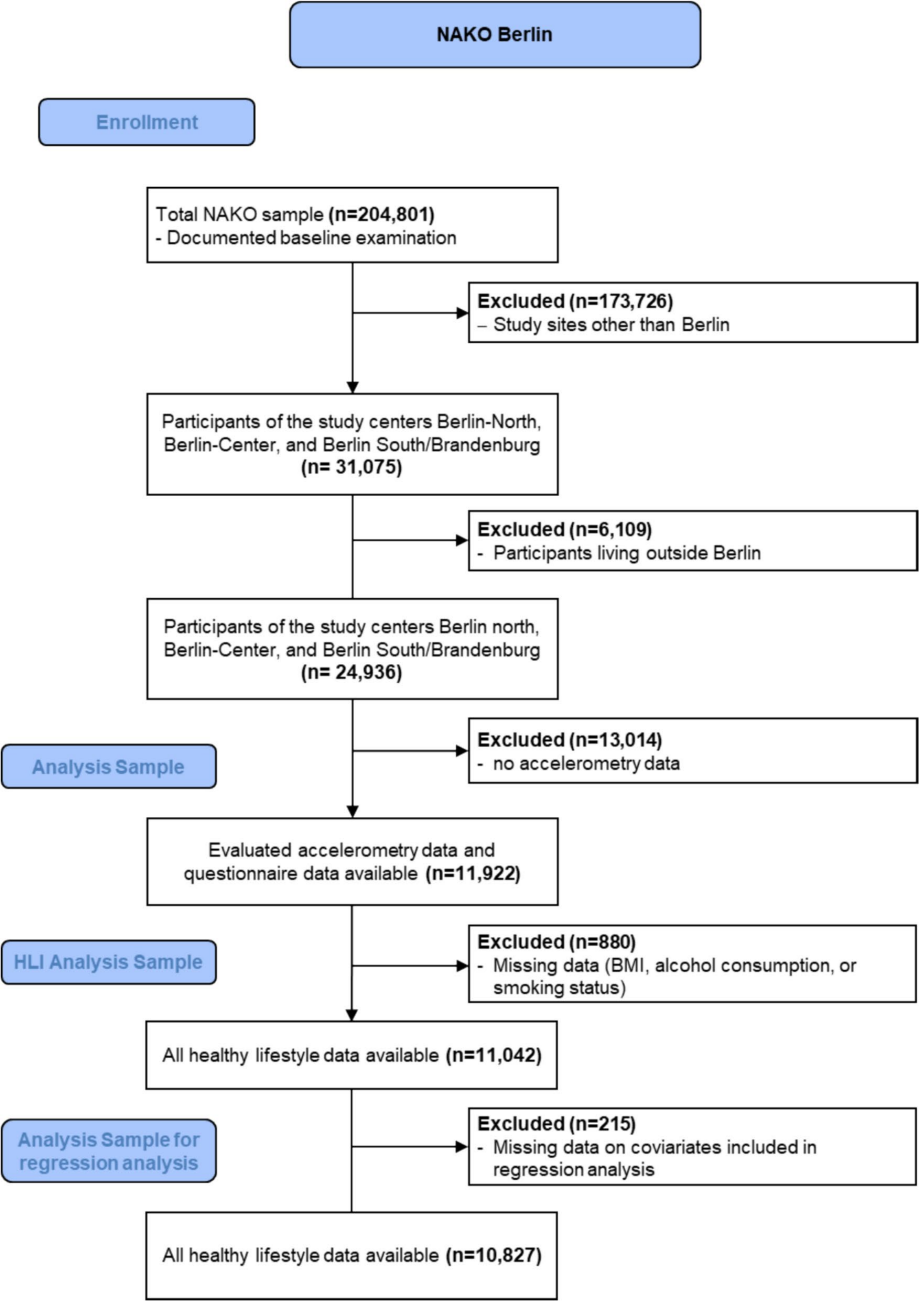
Recruitment flowchart

Out of over 200,000 NAKO participants, 31,075 were recruited in the three Berlin study centers. Out of those, 24,936 lived in Berlin, whereas the remaining were residents in the surrounding state of Brandenburg. Accelerometry data of participants living in Berlin were available from 11,922 participants who were included in our analyses (Fig. 2).

The mean age ± standard deviation (SD) of all subjects in the final analysis sample was 50.6 ± 12.9 years, and 52.9% were women. About half of the participants lived in prognosis areas with a good nSES (i.e. Social Index 1–3). From the best (Social Index 1) to the worst (Social Index 7) nSES, we observed a decline in age, education years and income, and an increase in the proportions of persons with Turkish descent and persons without a partner. There were differences regarding employment status, but without a linear trend, however, the proportion of unemployed participants was highest in the two lowest ranked prognosis areas. The proportion of men and women was similar across the prognosis areas. BMI and the proportion of smokers increased with worse nSES. No pattern could be observed for physical activity or alcohol consumption. Further details are described in Table 1. Characteristics of all participants (including also participants without accelerometry) did not differ from those of the accelerometry subsample (Supplementary Table 2).

**Table 1:** Characteristics of the study sample stratified by neighborhood SES and the total sample (*n* = 11,922)

		Total	Neighborhood SES						
			1 (best)	2	3	4	5	6	7 (worst)
		11,922	1127 (9.5)	2608 (21.9)	1989 (16.7)	2715 (22.8)	1581 (13.3)	1028 (8.6)	874 (7.3)
Number of prognosis areas		53	8	8	7	7	8	8	7
	N	N (%) or mean ± SD							
Sex	11,922								
Male		5627 (47.2)	541 (48.0)	1167 (44.7)	995 (50.0)	1215 (44.8)	773 (48.9)	506 (49.2)	430 (49.2)
Female		6295 (52.8)	586 (52.0)	1441 (55.3)	994 (50.0)	1500 (55.2)	808 (51.1)	522 (50.8)	444 (50.8)
Age	11,922	50.6 ± 12.9	51.8 ± 11.7	52.1 ± 11.9	49.1 ± 12.5	51.6 ± 12.4	49.4 ± 12.8	49.1 ± 13.9	49.3 ± 13.6
Education (ISCED 97)^1^	11,693								
High		7618 (65.2)	786 (70.4)	1833 (71.3)	1339 (69.9)	1651 (61.7)	993 (64.4)	541 (54.0)	457 (56.2)
Middle		3807 (32.6)	317 (28.4)	710 (27.6)	563 (29.0)	951 (35.5)	513 (33.2)	420 (42.0)	333 (39.4)
Low		268 (2.3)	13 (1.2)	27 (1.1)	40 (2.1)	74 (2.8)	37 (2.4)	40 (4.0)	37 (4.4)
Education years		15.9 (2.6)	16.3 (2.3)	16.3 (3.1)	16.2 (2.4)	15.7 (2.5)	15.9 (2.4)	15.2 (2.4)	15.2 (2.7)
Range of the means of education years within the prognosis areas		4.5–16.5	12.8–16.5	12.7–15.1	12.4–15.0	13.2–15.0	11.4–15.3	4.5–15.1	9.4–15.3
Employment status	11,826								
Employed		9099 (76.9)	866 (77.3)	2029 (78.2)	1607 (81.5)	2032 (75.4)	1214 (77.7)	716 (70.5)	635 (73.6)
Not employed		392 (3.3)	24 (2.1)	70 (2.7)	60 (3.0)	82 (3.0)	49 (3.1)	62 (6.1)	45 (5.2)
Economically inactive (retired or other reasons)		2335 (19.7)	231 (20.6)	497 (19.1)	304 (15.4)	582 (21.6)	300 (19.2)	238 (23.4)	183 (21.2)
Monthly net equivalent household income in €^2^	11,271	2310 ± 1449	2674 ± 1534	2563 ± 1605	2501 ± 1630	2195 ± 1294	2177 ± 1314	1903 ± 1209	1761 ± 946
Marital status	11,899								
Living with partner		7775 (65.3)	844 (74.9)	1785 (68.6)	1289 (65.0)	1714 (63.3)	983 (62.4)	641 (62.4)	519 (59.4)
Not living with partner		1327 (11.2)	90 (8.0)	256 (9.8)	231 (11.6)	313 (11.6)	204 (12.9)	126 (12.3)	107 (12.2)
No partner		2797 (23.5)	193 (17.1)	562 (21.6)	464 (23.4)	681 (25.1)	389 (24.7)	260 (25.3)	248 (28.4)
Turkish descent^3^	11,921	160 (1.3)	10 (0.9)	18 (0.7)	17 (0.9)	44 (1.6)	24 (1.5)	18 (1.8)	29 (3.3)
Lifestyle factors									
BMI^4^	11,580	25.8 ± 4.7	25.5 ± 4.5	25.5 ± 4.5	25.4 ± 4.3	25.8 ± 4.6	25.7 ± 4.5	26.4 ± 5.2	26.6 ± 5.5
Underweight		166 (1.4)	13 (1.2)	36 (1.4)	29 (1.5)	37 (1.4)	23 (1.5)	13 (1.3)	15 (1.8)
Normal weight		5554 (48.0)	545 (50.1)	1241 (49.4)	969 (49.8)	1236 (47.2)	758 (49.0)	439 (43.4)	366 (42.8)
Overweight		4005 (34.6)	376 (34.6)	856 (34.1)	689 (35.4)	919 (35.1)	525 (34.0)	355 (35.1)	285 (33.3)
Obesity		1855 (16.0)	154 (14.2)	378 (15.1)	260 (13.4)	428 (16.3)	240 (15.5)	205 (20.3)	190 (22.2)
Physical activity (MVPA-minutes per day)^5^	11,922	69.8 ± 34.2	68.5 ± 34.2	70.5 ± 33.7	70.9 ± 32.7	69.7 ± 34.6	70.5 ± 34.5	67.3 ± 35.2	68.0 ± 35.7
4th quartile (highest)		2987 (25.1)	260 (23.1)	645 (24.7)	536 (26.9)	701 (25.8)	405 (25.6)	232 (22.6)	208 (23.8)
3rd quartile		2973 (24.9)	274 (24.3)	677 (26.0)	499 (25.1)	641 (23.6)	423 (26.8)	246 (23.9)	213 (24.4)
2nd quartile		2984 (25.0)	289 (25.6)	681 (26.1)	504 (25.3)	683 (25.2)	362 (22.9)	260 (25.3)	205 (23.5)
1 st quartile (lowest)		2978 (25.0)	304 (27.0)	605 (23.2)	450 (22.6)	690 (25.4)	391 (24.7)	290 (28.2)	248 (28.4)
Smoking status	11,481								
Never		5179 (45.0)	550 (50.4)	1151 (45.8)	889 (46.2)	1145 (44.6)	641 (41.7)	460 (45.6)	343 (39.9)
Former		3871 (33.7)	370 (33.9)	894 (35.6)	639 (33.2)	879 (34.3)	511 (33.2)	297 (29.4)	281 (32.7)
Current		2431 (21.1)	169 (15.5)	463 (18.4)	396 (20.6)	537 (20.9)	381 (24.8)	251 (24.9)	234 (27.2)
Alcohol consumption (AUDIT-C Score)^6^	11,462								
0–3 (men)/0–2 (women)		5065 (44.2)	506 (46.5)	1035 (41.3)	858 (44.7)	1156 (45.2)	627 (41.0)	465 (46.2)	418 (48.8)
4–5 (men)/3–5 (women)		4684 (40.9)	449 (41.2)	1100 (43.9)	743 (38.7)	1060 (41.4)	647 (42.3)	375 (37.2)	310 (36.2)
6–7		1261 (11.0)	99 (9.1)	279 (11.1)	232 (12.1)	251 (9.8)	186 (12.2)	118 (11.7)	96 (11.2)
8–12		452 (3.9)	35 (3.2)	90 (3.6)	86 (4.5)	92 (3.6)	68 (4.5)	49 (4.9)	32 (3.7)
Risky alcohol consumption AUDIT-C Score ≥ 4 (men)/≥ 3 (women)	11,462	4074 (35.5)	361 (33.1)	938 (37.5)	679 (35.4)	884 (34.5)	590 (38.6)	341 (33.9)	281 (32.8)
Subjective health status	11,490								
Good, very good, excellent		7252 (63.1)	673 (61.5)	1538 (61.3)	1161 (60.2)	1659 (64.8)	955 (62.3)	667 (66.1)	599 (70.0)
Fair, poor		4238 (36.9)	421 (38.5)	973 (38.7)	766 (39.8)	900 (35.2)	579 (37.7)	342 (33.9)	257 (30.0)
Life satisfaction (0 = worst, 10 = best)	11,493	7.6 ± 1.9	7.9 ± 1.8	7.7 ± 1.8	7.7 ± 1.8	7.5 ± 2.0	7.6 ± 1.9	7.6 ± 2.0	7.6 ± 1.9

^1^ISCED97: International standard classification of education [38]

^2^Net household income divided by corresponding household members

^3^Turkish descent, if i) own Turkish background or ii) migration of at least one parent from Turkey to Germany after 1949 [36]

^4^BMI: Body Mass index in kg/m^2^; underweight: < 18.5 kg/m^2^, normal weight: 18.5–24.9 kg/m^2^, overweight: 25.0–29.9 kg/m^2^, obesity: > 30.0 kg/m^2^)

^5^MVPA: moderate-to-vigorous physical activity

^6^AUDIT-C: The AUDIT Alcohol Consumption Questions [33]

### Healthy Lifestyle Index

The individual mean ± SD HLI was 8.3 ± 2.0 out of a maximum of 12 points. Of all participants, 4%, 44.1%, 42.4%, 9.1%, and 0.4% engaged in four, three, two, one, or no beneficial health behaviors, respectively. A small trend could be observed for a slightly higher HLI in prognosis areas with a better nSES compared to those with a worse nSES (Table 2). The proportion of persons with normal BMI (< 25 kg/m2) and beneficial smoking behavior (never smoking) decreased alongside the nSES, while the differences in alcohol consumption and physical activity did not show any trend.

**Table 2:** Healthy Lifestyle Index and individual lifestyle factors for the total sample and stratified by nSES

		Neighborhood SES						
	Total (N = 11,042)	1 (best)	2	3	4	5	6	7 (worst)
	mean ± SD or n (%)							
Healthy Lifestyle Index (0 = worst, 12 = best score)	8.3 ± 2.0	8.4 ± 1.9	8.3 ± 2.0	8.4 ± 2.0	8.3 ± 2.0	8.2 ± 2.1	8.0 ± 2.1	8.0 ± 2.2
Highest Score achieved for								
Normal BMI^1^ (< 25 kg/m2)	5720 (49.4)	558 (51.3)	1277 (50.9)	998 (51.3)	1273 (48.6)	781 (50.5)	452 (44.7)	381 (44.5)
Physical activity^2^	2987 (25.1)	260 (23.1)	645 (24.7)	536 (26.9)	701 (25.8)	405 (25.6)	232 (22.6)	208 (23.8)
Never-smokers	5179 (45.5)	550 (50.9)	1151 (46.1)	889 (46.8)	1145 (45.2)	641 (42.3)	460 (45.9)	343 (40.4)
Low risk alcohol consumption^3^	5065 (44.2)	506 (46.5)	1035 (41.3)	858 (44.7)	1156 (45.2)	627 (41.0)	465 (46.2)	418 (48.8)
Number of beneficial health behaviors (max. 4 out of 4)	2.4 ± 0.7	2.5 ± 0.7	2.4 ± 0.7	2.5 ± 0.7	2.4 ± 0.7	2.4 ± 0.7	2.3 ± 0.8	2.3 ± 0.8
4 (HLI = 12)	442 (4.0)	47 (4.5)	97 (4.0)	80 (4.3)	102 (4.2)	61 (4.1)	30 (3.0)	25 (3.0)
3	4864 (44.1)	482 (46.1)	1086 (45.3)	858 (46.2)	1088 (44.6)	631 (42.7)	392 (39.8)	327 (39.4)
2	4677 (42.4)	445 (42.6)	1007 (42.0)	768 (41.3)	1028 (42.1)	627 (42.5)	436 (44.3)	366 (44.1)
1	1006 (9.1)	69 (6.6)	202 (8.4)	148 (8.0)	214 (8.8)	150 (10.2)	121 (12.3)	102 (12.3)
0	47 (0.4)	2 (0.2)	7 (0.3)	4 (0.2)	10 (0.4)	8 (0.5)	6 (0.6)	10 (1.2)

^1^BMI: Body mass index

^2^upper quartile of sample

^3^AUDIT-C: < 4 (men), < 3 (women)

### Characterization of the Berlin prognosis areas

**Fig. 3:**
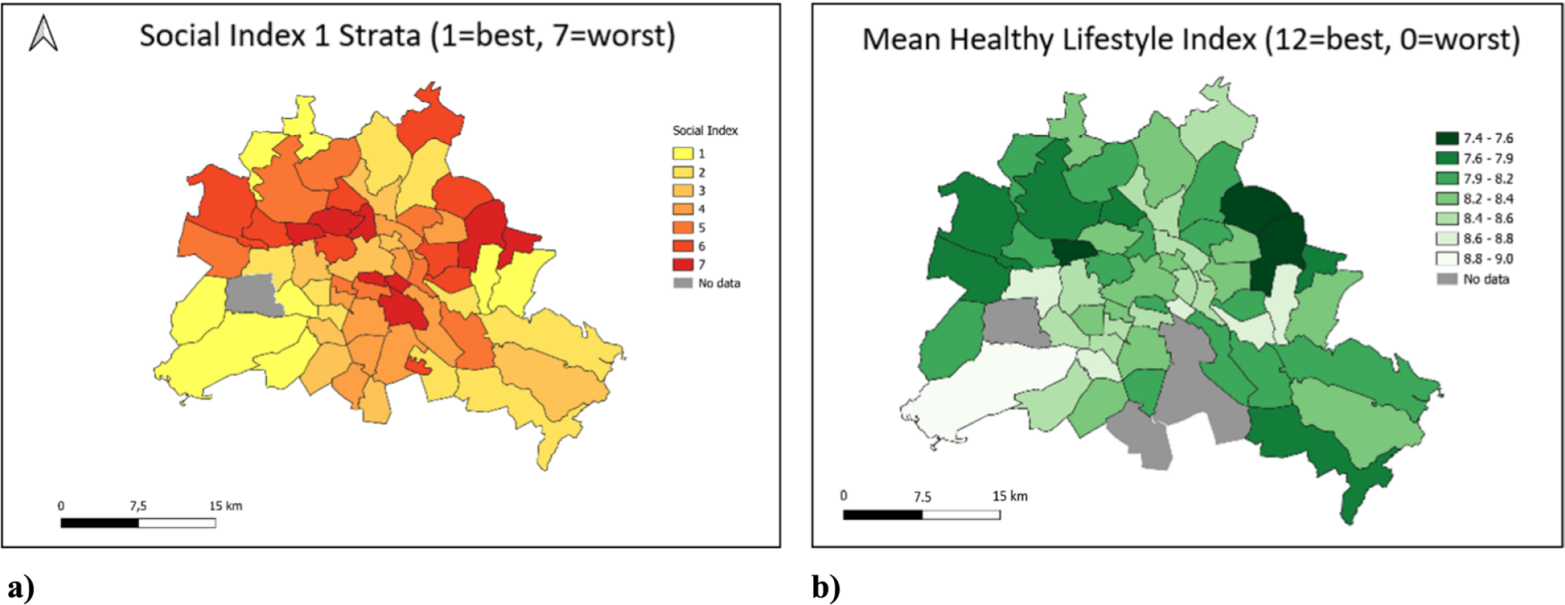
Prognosis areas of Berlin with (**a**) neighborhood socioeconomic status represented by the Social Index of the Sozialstrukturatlas 2013 and (**b**) the Healthy lifestyle index (HLI). The HLI values indicate the average HLI of all NAKO participants living in the respective prognosis areas. Grey shaded areas indicate prognosis areas with less than 20 participants

**Fig. 4:**
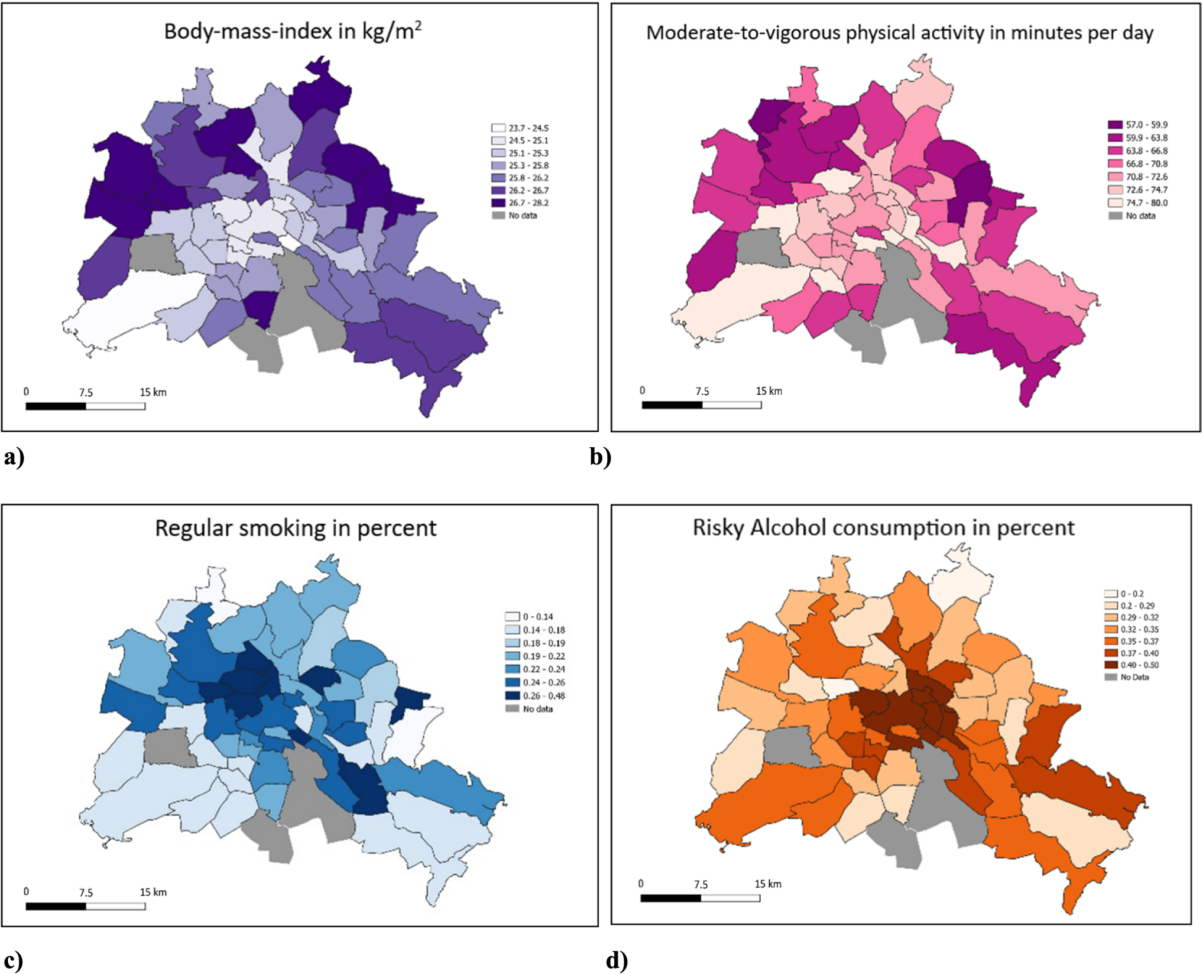
Prognosis areas of Berlin showing (**a**) mean body mass index in kg/m^2^ (BMI), (**b**) moderate-to-vigorous physical activity in minutes per day, (**c**) proportion of regular smoking, and (**d**) proportion of risky alcohol consumption. The values indicate the average values of all NAKO participants living in the respective prognosis areas. Grey shaded areas indicate prognosis areas with less than 20 participants

The heatmaps in Fig. 3 show a) the original figure of the social index (adapted from the Sozialstrukturatlas 2013) and b) the HLI among the 53 prognosis areas with sufficient data from this analysis. The individual health behaviors are shown in the following heatmaps represented by Fig. 4a-d. Beneficial health behaviors are shown in light colors that gradually darken as behaviors become less beneficial. In the overlap of the Social Structure Atlas and the HLI, there is a trend toward spatial concordance across several prognosis areas; however, certain areas deviate from this trend.

Among individual health behaviors, BMI was lowest in the central prognosis areas and in the affluent southwest. Physical activity followed a relatively similar spatial pattern. By contrast, the occurrence of smoking was lowest in more decentralized areas with lower population density and abundant green space, rather than in the city center. Alcohol consumption, meanwhile, was relatively heterogeneous across all prognosis areas but showed a clear cluster of higher intake in the central districts (Fig. 4).

### Association of neighborhood SES with Healthy Lifestyle Index

In the multivariable regression analysis each one-unit worsening in the Social Index was associated with a 0.08-point reduction in HLI, indicating a very small association of nSES with a healthy lifestyle. This effect could be shown independently of the individual SES (represented by education status). Among the covariates, only education as surrogate for individual socioeconomic status showed an association with HLI (adjusted beta coefficient (95% CI) −1.29 (−1.56; −1.01) for low compared to high education). Among the individual health behaviors, there were associations of BMI and smoking (4% and 5% lower proportion of normal weight and never smoking per one-unit worsening of the Social Index, respectively) with nSES, while there was no association of alcohol consumption or physical activity with nSES (Table 3). To explore whether the association might deviate from strict linearity, we additionally modelled nSES using the seven original Social Index categories (category 1 as reference; Table 3). These category-specific estimates suggest a modest stepwise decline, with smaller differences across categories 2–4 (around − 0.10) and a more pronounced drop from category 5 onwards (approximately − 0.30 in category 5, − 0.44 in category 6, and − 0.49 in category 7 vs category 1). Taken together, the continuous model summarizes the overall trend as a small average linear decrease, while the categorical model indicates that differences may be concentrated in the more disadvantaged categories rather than evenly distributed across all categories. For the individual lifestyle factors (logistic regression), a one-unit worsening in the Social Index was associated with lower odds of normal weight (adjusted OR 0.95, 95% CI 0.93–0.97) and lower odds of never smoking (adjusted OR 0.96, 95% CI 0.94–0.98), whereas associations with upper-median physical activity and no risky alcohol consumption were close to null (adjusted ORs around 1.00, Table 3). In the seven-category models, the clearest pattern again appeared in the more disadvantaged categories (particularly categories 5–7), where the odds of normal weight and never smoking tended to be lower compared with category 1, while results for physical activity and alcohol showed no consistent gradient (Table 3). Detailed univariate and multivariable results are presented in Supplementary Table 3.

**Table 3:** Association of nSES with Healthy Lifestyle Index and all four lifestyle factors presented as multivariable linear (outcome: Healthy Lifestyle Index HLI) and logistic (outcomes: normal weight, upper median of physical activity (PA), never smoking, no risky alcohol consumption vs respective counterparts) regression analyses; *N* = 10,827

	Healthy Lifestyle Index	Normal weight	Upper median PA	Never smoking	No risky alcohol consumption
	Adjusted* beta coefficient (95% CI)	Adjusted* OR (95% CI)			
Social index (per unit worsening)	−0.08 (−0.10; −0.06)	0.95 (0.93;0.97)	0.98 (0.96; 1.01)	0.96 (0.94; 0.98)	1.00 (0.98; 1.03)
Social Index categories 1	Ref	Ref	Ref	Ref	Ref
2	−0.10	0.96 (0.83; 1.12)	1.18 (1.02; 1.37)	0.82 (0.71; 0.95)	0.83 (0.72; 0.97)
3	−0.11	0.91 (0.78; 1.06)	1.11 (0.95; 1.29)	0.82 (0.70; 0.96)	0.93 (0.79; 1.09)
4	−0.10	0.89 (0.77; 1.04)	1.14 (0.99; 1.32)	0.82 (0.71; 0.95)	0.92 (0.79; 1.07)
5	−0.30	0.90 (0.77; 1.06)	1.15 (0.98; 1.36)	0.70 (0.60; 0.82)	0.78 (0.66; 0.92)
6	−0.44	0.72 (0.60; 0.87)	0.93 (0.78; 1.11)	0.86 (0.72; 1.03)	0.95 (0.79; 1.15)
7	−0.49	0.71 (0.59; 0.87)	1.0 (0.83; 1.21)	0.67 (0.56; 0.81)	1.0 (0.82; 1.21)

^*^Adjusted for sex, age, Turkish descent, and education

As an additional descriptive contrast, we collapsed the Social Index into higher (categories 1–4) vs lower (categories 5–7) nSES. In this comparison, lower nSES (5–7) was associated with a − 0.30-point lower HLI (95% CI − 0.38 to − 0.22) and with lower odds of normal weight (OR 0.86, 95% CI 0.79–0.94) and never smoking (OR 0.88, 95% CI 0.80–0.95), while associations remained weak for physical activity (OR 0.93, 95% CI 0.85–1.01) and no risky alcohol consumption (OR 0.97, 95% CI 0.89–1.06) (Supplementary Table 4). We further explored whether these associations differed by sex by conducting sex-stratified analyses and formally testing effect modification using a Social Index × sex interaction term in pooled models. Sex-stratified estimates were broadly similar for physical activity, never smoking, and no risky alcohol consumption, and interaction tests did not provide clear evidence for heterogeneity (p-interaction = 0.481, 0.203, and 0.055, respectively). In contrast, we observed evidence of effect modification for normal weight (p-interaction = 0.002): lower nSES was more strongly associated with lower odds of normal weight among women (OR 0.76, 95% CI 0.67–0.85) than among men, where the association was essentially null (OR 0.99, 95% CI 0.87–1.12) (Supplementary Table 5). There was no evidence of effect modification for the interaction between neighborhood socioeconomic status (nSES) and individual socioeconomic status (SES), *p* > 0.999).

## Discussion

### Main findings

This is, to our knowledge, the first study that investigated the association of neighborhood socioeconomic status with partly objectively measured health behaviors (combined as Healthy Lifestyle Index (HLI) as well as for each individual factor) in Berlin. The mean Healthy Lifestyle Index of the participants was 8.3 ± 2.0 out of 12 achievable points. Only 4% of the participants engaged in all four benefical health behaviors with only small differences regarding nSES. This observation was confirmed by regression analyses showing only a small association between nSES and healthy lifestyle in Berlin, adjusted for individual SES and other sociodemographic covariates. In practical terms, the estimated − 0.08-point difference in HLI per one-category worsening in the Social Index is very small (< 1% change on the 0–12 scale) and far below a one-point change, which would reflect a meaningful shift in at least one component. Among the four evaluated health behaviors, BMI and smoking were associated with nSES, whereas physical activity and alcohol consumption were not.

When we used nSES as categorical variable with its seven original categories, the differences in HLI were small between categories 1–4, but the decrease was stronger in the more disadvantaged categories (5–7), which suggests more a threshold pattern than a fully linear trend. In line with this, in the dichotomous analysis (categories 1–4 vs. 5–7), lower nSES was associated with a lower mean HLI and lower odds of normal weight and never smoking, while the associations with physical activity and risky alcohol consumption remained weak and not statistically significant. In sex-stratified analyses, we found evidence of effect modification for normal weight: the inverse association between lower nSES and normal weight was more pronounced among women, whereas it was close to null among men (p-interaction = 0.002). The described associations were partly visible in the maps. While BMI, smoking behavior and to a certain extent physical activity showed similar patterns on the maps like the HLI, alcohol consumption seemed to be driven less by nSES as an aggregate measure but more by single or spatially clustered prognosis areas in which favorable or unfavorable behaviors concentrate.

### Comparison with other studies

The findings of our study integrate partly into the broader evidence on neighbourhood context and lifestyle. A consistent observation across high-income countries is that smoking and excess body weight rise with area deprivation. This pattern has been documented for German and Czech cities [39], across multiple European settings [16], and in US national samples [40]. A recent meta-analysis confirmed the association of nSES with overweight and obesity for Australia, the US, and Europe for both children and adults [41]. Similar to our findings, other authors have reported that the association between neighborhood SES and BMI is more pronounced among women than among men [42]. Our Berlin data are in line with these associations for smoking and BMI, independent of individual SES. The most important explanation is that the social structure atlas includes smoking behavior. The observed association with nSES was therefore expected and can be interpreted as confirmation of the construct. In addition, an environmental explanation might be plausible: a study conducted in a large city in Germany showed denser clusters of tobacco, alcohol and fast-food outlets in deprived districts [43]. In contrast, a Dutch study using data from the large national Lifelines cohort suggested that nSES itself, rather than fast food-outlet density, explained most of the BMI excess in deprived areas [44]. Since we did not look deeper into the food environment of the prognosis areas, the causal pathway for Berlin remains unclear.

In contrast to smoking behavior and BMI, we found no association—neither crude nor adjusted—between nSES and objectively measured MVPA. This result is in line with an accelerometer study in older UK residents in which deprivation effects disappeared after adjustment for individual education and physical function [45]. It diverges, however, from the longitudinal US CARDIA cohort, which reported lower activity (measured by self-report) in deprived areas, particularly among black adults [46]. Possible explanations might be i) Berlin’s public-transport infrastructure and abundant green space which equalise opportunities for physical activity across districts, as well as ii) the fact that accelerometers capture total movement—including domestic, transport, and work domains—which are less socio-economically patterned than leisure-time sport typically measured in questionnaires.

NSES was likewise unrelated to risky alcohol consumption, in line with the null or mixed findings in the European systematic review by Algren et al. [16] and a study from the US exploring the association of nSES with alcohol and drug use in adults [40]. One explanation for this result might be the ubiquitous alcohol retail in Germany—and especially in Berlin with its high number of convenience shops that stay open very late or even all night, selling cold drinks including alcohol, snacks, and everyday essentials when regular stores are closed. Furthermore, the relatively homogeneous drinking norms may attenuate spatial variation in consumption. Unlike smoking, alcohol consumption in Germany is less regulated and widespread across all social strata [47]. Finally, studies investigating co-occurring behaviours operationalised by a lifestyle index showed that unhealthy habits cluster in low-SES environments (Ortiz et al. 2022). We similarly observed that multiple beneficial behaviours accumulate mainly in a few advantaged prognosis areas, highlighting micro-level hotspots more than a uniform SES gradient [48].

### Strengths and limitations

A key strength of our investigation was the large, population-based sample including persons living across 53 of 60 defined neighbourhoods in Berlin. By linking geocoded residential addresses of the study participants to the Berlin Social-Structure Atlas, we were able to examine contextual deprivation while simultaneously adjusting for a variety of individual sociodemographic characteristics. Another advantage is the comprehensive lifestyle assessment: BMI was measured by trained staff, and physical activity was captured objectively with hip-worn accelerometers—an approach that avoids the recall bias inherent in self-reported activity. The construction of a four-component healthy-lifestyle index further enabled us to study behavioural clustering rather than isolated lifestyle behaviors.

Several limitations should be acknowledged. First, the cross-sectional design precludes causal inference and cannot capture residential mobility or length of exposure. Therefore, we cannot determine whether low nSES leads to unhealthy behaviour or whether people with less healthy lifestyles are more likely to reside in deprived areas. Participants are clustered within prognosis areas; because we used single-level regression models, standard errors may be somewhat underestimated if within-area correlation is present. Future analyses could use multilevel models or cluster-robust standard errors to quantify the impact of within-area correlation. Also, effect estimates were relatively small and might have limited clinical relevance, even if statistically significant. Second, the Social Index we used is aggregated at the prognosis-area level; smaller spatial units might detect stronger contextual effects. However, due to data protection reasons we decided to include only prognosis areas with at least 20 participants in our analyses. Also, the number of participants for the included prognosis areas differed ranging from 21 to 1037 participants. Third, selection bias is possible because the overall NAKO response rate was about 17%; health-conscious individuals as well as persons with higher individual socioeconomic status were over-represented, potentially attenuating contextual differences. Dichotomising behaviours improves interpretability and alignment with the HLI scoring, but may reduce sensitivity to dose–response patterns; future work could examine PA and alcohol using continuous or ordinal models. Additionally, there was only information on Turkish migration background available without the possibility to distinguish between first and second generation migrants. Finally, unmeasured contextual characteristics— walkability, green-space quality, safety, social cohesion— were not captured in the Social Index and could confound or mediate the modest associations observed (Supplementary Fig. 2). However, given Berlin’s extensive and widely distributed green space and mixed land use, it is unlikely that our behaviour patterns merely reflect an inner–periphery (urban–rural) gradient [49].

### Public health implications

Our study is based on cross-sectional analyses, thus our results cannot be interpreted causally and should be viewed as descriptive information of co-occurring patterns. Nevertheless, they may offer useful signals for public health planning in Berlin. Most notably, the mean HLI was well below the maximum of 12 points also in areas with high nSES. This suggests a considerable scope to promoting a healthy lifestyle across all districts and prognosis areas for all of the four health behaviors underlying the HLI. Across these behaviors, the spatial patterns indicate that priorities may differ by area, but any targeted action should be framed as hypothesis-generating rather than definitive. Since area deprivation was linked to higher smoking prevalence and higher BMI, interventions could apply intensified tobacco control and healthy-weight promotion in these disadvantaged prognosis areas. Possible measures could include stricter enforcement of smoke-free housing rules, tighter licensing of tobacco vendors, and improved access to affordable healthy foods. The distribution of alcohol consumption suggests that interventions should be planned with local context in mind (inner-city districts) while maintaining citywide reach for measures regarding physical activity promotion. Our spatial distribution maps can help to support this planning. Finally, since individual SES, measured by education, showed the strongest association with a healthy lifestyle, strategies that combine structural changes with person-centred approaches appear particularly relevant.. Strengthening health literacy starting early in life (e.g. in kindergarten and in school settings) and continuing through adulthood may help mitigate disparities associated with parental education and promote equal educational opportunities for all Berliners. Additionally, longitudinal and intervention research is needed to test whether these approaches lead to measurable improvements in HLI over time.

## Conclusions

The association of low neighborhood socioeconomic status (nSES) and the Healthy Lifestyle Index (HLI) in Berlin was small yet persisted after adjustment. Among the individual lifestyle behaviors, lower nSES was associated with slightly less favourable body weight and a modestly higher smoking share, but showed no associations with risky alcohol consumption and objectively measured physical activity. Differences in HLI and related behaviours appeared to be mainly driven by the most disadvantaged nSES groups. While our maps suggest that lifestyle factors may cluster in a few discrete prognosis areas rather than follow a uniform nSES gradient, this pattern is based on visual inspection only and should be treated as hypothesis-generating. Future studies should examin spatial clustering more formally and use longitudinal data to account for neighborhood residence duration and to generate more robust evidence on these associations. Additionally, the built environment and access to green spaces should be considered, in order to enable a more comprehensive understanding of the complex interplay between contextual factors and their influence on health-related behaviors and lifestyle.

## Supplementary Information

Supplementary Material 1. Supplementary Table 1. Calculation of Healthy Lifestyle Index (HLI). Supplementary Table 2. Characteristics of the study sample stratified by neighborhood SES and the total sample (*n* = 24,936) including also participants without accelerometry. Supplementary Table 3. Multivariable linear (outcome: Healthy Lifestyle Index HLI) and logistic (outcomes: normal weight, upper median of physical activity (PA), never smoking, no risky alcohol consumption vs respective counterparts) univariate and multivariable regression analyses; multivariable analyses: *N* = 10,827; adjusted for sex, age, Turkish descent, and education. Supplementary Table 4. Multivariable linear (outcome: Healthy Lifestyle Index HLI) and logistic (outcomes: normal weight, upper median of physical activity (PA), never smoking, no risky alcohol consumption vs respective counterparts) regression analyses; multivariable analyses: N=10,827; adjusted for sex, age, Turkish descent, and education. Supplementary Table 5. Sex-stratified multivariable linear (outcome: Healthy Lifestyle Index HLI) and logistic (outcomes: normal weight, upper median of physical activity (PA), never smoking, no risky alcohol consumption vs respective counterparts) regression analyses; multivariable analyses: *N* = 10,827; adjusted for sex, age, Turkish descent, and education. Supplementary Figure 1. Association of the Mean Healthy Lifestyle Index (± 1 standard deviation) with nSES. Supplementary Figure 2. Conceptual framework of nSES, covariates and potential environmental confounders/mediators with Healthy Lifestyle Index.

## Data Availability

The datasets used and/or analysed during the current study are available from the corresponding author on reasonable request.

## Abbreviations

AUDIT-C: Alcohol Use Disorders Identification Test – Consumption
BMI: Body mass index
CI: Confidence interval
GISD: German Index of Socioeconomic Deprivation
HLI: Healthy lifestyle index
ISCED: International Standard Classification of Education
kg: Kilogram
m: Meter
MAD: Mean amplitude deviation
*mg*: Milli gravity
MVPA: Moderate-to-vigorous physical activity
NAKO: German National Cohort Study
nSES: Neighborhood socioeconomic status
OR: Odds ratio
SD: Standard deviation
SES: Socioeconomic status

## References

[1] Hoebel J, Müters S. Sozioökonomischer Status und Gesundheit. WSI Mitt. 2024;77(3):172–9. 10.5771/0342-300X-2024-3-172.

[2] Kim Y, Vazquez C, Cubbin C. Socioeconomic disparities in health outcomes in the United States in the late 2010s: results from four national population-based studies. Arch Public Health. 2023;81(1):1–10. 10.1186/s13690-023-01026-1. 36739440 10.1186/s13690-023-01026-1 PMC9899106

[3] Ross C, Wu C. The Links Between Education and Health Author ( s ): Catherine E . Ross and Chia-ling Wu Published by : American Sociological Association Stable URL : http://www.jstor.org/stable/2096319 Accessed : 22-03-2016 12 : 47 UTC Your use of the JSTOR archive indi. Am Sociol Rev. 1995;60(5):719–45.

[4] Liu B, Ji S, Zhu Z. Does higher education matter for health in the UK? SSM Popul Health. 2024;25:101642. 10.1016/j.ssmph.2024.101642. 38440105 10.1016/j.ssmph.2024.101642 PMC10909631

[5] Viinikainen J, Bryson A, Böckerman P, Kari JT, Lehtimäki T, Raitakari O, et al. Does better education mitigate risky health behavior? A mendelian randomization study. Econ Hum Biol. 2022;46:0–2. 10.1016/j.ehb.2022.101134. 10.1016/j.ehb.2022.101134 35354116

[6] You Y, Mo L, Tong J, Chen X, You Y. The role of education attainment on 24-hour movement behavior in emerging adults: evidence from a population-based study. Frontiers in Public Health. 2024;12:1197150. 10.3389/fpubh.2024.1197150. 38292911 10.3389/fpubh.2024.1197150 PMC10824836

[7] Blakely T, Hunt D, Woodward A. Confounding by socioeconomic position remains after adjusting for neighbourhood deprivation: an example using smoking and mortality. J Epidemiol Community Health. 2004;58(12):1030–1. 10.1136/jech.2004.019737. 15547067 10.1136/jech.2004.019737 PMC1732651

[8] Hoebel AJ, Michalski N, Baumert J, Nowossadeck E, Tetzlaff F. The life expectancy gap : Socioeconomic differences in life expectancy between areas in Germany. J Health Monit. 2025;10(1):1–8. 10.25646/13026. 10.25646/13026 PMC11948288 40161013

[9] Jivraj S, Murray ET, Norman P, Nicholas O. The impact of life course exposures to neighbourhood deprivation on health and well-being: a review of the long-term neighbourhood effects literature. Eur J Public Health. 2020;30(5):922–8. 10.1093/EURPUB/CKZ153. 31576400 10.1093/eurpub/ckz153 PMC8489013

[10] Diez Roux AV, Mair C. Neighborhoods and health. Ann N Y Acad Sci. 2010;1186:125–45. 10.1111/j.1749-6632.2009.05333.x. 20201871 10.1111/j.1749-6632.2009.05333.x

[11] Pickett KE, Pearl M. Multilevel analyses of neighbourhood socioeconomic context and health outcomes : a critical review. Rev J Epidemiol Community Health. 2007;55(2):111–22. 10.1136/jech.55.2.111 PMC1731829 11154250

[12] Stafford M, Marmot M. Neighbourhood deprivation and health: does it affect us all equally? Int J Epidemiol. 2003;32(3):357–66. 10.1093/ije/dyg084. 12777420 10.1093/ije/dyg084

[13] Malkowski OS, Harvey J, Townsend NP, Kelson MJ, Foster CEM. Correlates and determinants of physical activity among older adults of lower versus higher socio‑economic status: a systematic review and meta‑analysis. Int J Behav Nutr Phys Act. 2025;22(83):1–16. 40545526 10.1186/s12966-025-01775-y PMC12183859

[14] Ribeiro AI, Fraga S, Severo M, Kelly-Irving M, Delpierre C, Stringhini S, et al. Association of neighbourhood disadvantage and individual socioeconomic position with all-cause mortality: a longitudinal multicohort analysis. The Lancet Public Health. 2022;7(5):e447–57. 10.1016/S2468-2667(22)00036-6. 35487230 10.1016/S2468-2667(22)00036-6

[15] Zhang K, Lovasi GS, Odden MC, Michael YL, Newman AB, Arnold AM, et al. Association of retail environment and neighborhood socioeconomic status with mortality among community-dwelling older adults in the United States: Cardiovascular Health Study. J Gerontol: Series A. 2022;77(11):2240–7. 10.1093/gerona/glab319. 10.1093/gerona/glab319 PMC9678200 34669918

[16] Algren MH, Bak CK, Berg-Beckhoff G, Andersen PT. Health-risk behaviour in deprived neighbourhoods compared with non-deprived neighbourhoods: a systematic literature review of quantitative observational studies. PLoS ONE. 2015;10(10):1–17. 10.1371/journal.pone.0139297. 10.1371/journal.pone.0139297 PMC4624433 26506251

[17] Dittmann J, Goebel J. Your house, your car, your education: the socioeconomic situation of the neighborhood and its impact on life satisfaction in Germany. Soc Indic Res. 2010;96(3):497–513. 10.1007/s11205-009-9489-7.

[18] Müller G, Berger K. Neighbourhood deprivation and type 2 diabetes: Results from the dortmund health study (DHS) [Der Zusammenhang von Deprivation im Wohnumfeld und der Typ-2-Diabetes- Prävalenz: Ergebnisse der Dortmunder Gesundheitsstudie (Do-GS)]. Gesundheitswesen. 2013;75(12):797–802. 23487321 10.1055/s-0033-1333737

[19] Voigtländer S, Berger U, Razum O. The impact of regional and neighbourhood deprivation on physical health in Germany: a multilevel study. BMC Public Health. 2010. 10.1186/1471-2458-10-403. 20615214 10.1186/1471-2458-10-403 PMC2912812

[20] Kroll LE, Schumann M, Hoebel J, Lampert T. Regional health differences-developing a socioeconomic deprivation index for Germany. J Health Monit. 2017;2(2):98–114. 10.17886/RKI-GBE-2017-048.2. 37152089 10.17886/RKI-GBE-2017-048.2 PMC10161274

[21] Michalski N, Reis M, Tetzlaff F, Herber M, Kroll LE, Hövener C, et al. German index of socioeconomic deprivation (GISD): revision, update and applications. J Health Monit. 2022;7(S5):1–120. 10.25646/10641. 10.25646/10641 PMC9768633 36628258

[22] Hoebel J, Nowossadeck E, Michalski N, Baumert J, Wachtler B, Tetzlaff F. Socioeconomic deprivation and premature mortality in Germany, 1998–2021: an ecological study with what-if scenarios of inequality reduction. Bundesgesundheitsblatt Gesundheitsforschung Gesundheitsschutz. 2024;67(5):528–37. 10.1007/s00103-024-03862-0. 38587641 10.1007/s00103-024-03862-0 PMC11093858

[23] Moissl AP, Delgado GE, Krämer BK, März W, Kleber ME, Grammer TB. Area-based socioeconomic status and mortality: the Ludwigshafen Risk and Cardiovascular Health study. Clin Res Cardiol. 2020;109(1):103–14. 10.1007/s00392-019-01494-y. 31144063 10.1007/s00392-019-01494-y

[24] Tetzlaff F, Nowossadeck E, Jansen L, Michalski N, Barnes B, Kraywinkel K, et al. Widening area-based socioeconomic inequalities in cancer mortality in Germany between 2003 and 2019. Sci Rep. 2023;13(1):1–11. 10.1038/s41598-023-45254-5. 37857781 10.1038/s41598-023-45254-5 PMC10587166

[25] Zimmermann J. Impact of neighborhood context on self-rated health among very old adults living in Germany: a cross-sectional representative study. BMC Geriatr. 2024;24(1):1–10. 10.1186/s12877-024-05175-y. 38969988 10.1186/s12877-024-05175-y PMC11227241

[26] Eurostat. Area by NUTS 3 region - 2015. Area by NUTS 3 Region - 2015. 2020. https://ec.europa.eu/eurostat/databrowser/view/demo_r_d3area/default/table?lang=en.

[27] Eurostat. Regions in Europe. Population on 1 January 2023. 2023. https://ec.europa.eu/eurostat/de/web/interactive-publications/regions-2024.

[28] Wikipedia. Demographics of the European Union - Wikipedia. 2025. https://en.wikipedia.org/wiki/Demographics_of_the_European_Union.

[29] Senatsverwaltung für Gesundheit und Soziales. Handlungsorientierter Sozialstrukturatlas Berlin 2013. 2014.

[30] Peters A, Peters A, Greiser KH, Göttlicher S, Ahrens W, Albrecht M, et al. Framework and baseline examination of the German National Cohort (NAKO). Eur J Epidemiol. 2022;37(10):1107–24. 10.1007/s10654-022-00890-5. 36260190 10.1007/s10654-022-00890-5 PMC9581448

[31] Rach S, Sand M, Reineke A, Becher H, Greiser KH, Wolf K, et al. The baseline examinations of the German National Cohort (NAKO): recruitment protocol, response, and weighting. Eur J Epidemiol. 2025;40(4):475–89. 10.1007/s10654-025-01219-8. 40259125 10.1007/s10654-025-01219-8 PMC12145326

[32] Leitzmann M, Gastell S, Hillreiner A, Herbolsheimer F, Baumeister SE, Bohn B, Brandes M, Greiser H, Jaeschke L, Jochem C, Kluttig A, Krist L, Michels KB, Pischon T, Schmermund A, Sprengeler O, Zschocke J, Ahrens W, Baurecht H, Steindorf K. Physical activity in the German National Cohort (NAKO): use of multiple assessment tools and initial results. Bundesgesundheitsblatt - Gesundheitsforschung - Gesundheitsschutz. 2020;63(3):301–11. 10.1007/s00103-020-03099-7. 32055903 10.1007/s00103-020-03099-7

[33] Bush K, Kivlahan DR, McDonell MB, Fihn SD, Bradley KA. The AUDIT alcohol consumption questions (AUDIT-C). Arch Intern Med. 1998;158:1789–95. 9738608 10.1001/archinte.158.16.1789

[34] Weber A, van Hees VT, Stein MJ, Gastell S, Steindorf K, Herbolsheimer F, Ostrzinski S, Pischon T, Brandes M, Krist L, Marschollek M, Greiser KH, Nimptsch K, Brandes B, Jochem C, Sedlmeier AM, Berger K, Brenner H, Buck C, Baurecht H. Large-scale assessment of physical activity in a population using high-resolution hip-worn accelerometry: the German National Cohort (NAKO). Sci Rep. 2024;14(1):1–13. 10.1038/s41598-024-58461-5. 38575636 10.1038/s41598-024-58461-5 PMC10995156

[35] Wolf K, Schikowski T, Dallavalle M, Niedermayer F, Bolte G, Lakes T, et al. Environmental exposure assessment in the German National Cohort (NAKO). Eur J Public Health. 2023;33(Supplement_2):121259. 10.1093/eurpub/ckad160.501. 10.1016/j.envres.2025.121259 40023386

[36] Wiessner C, Keil T, Krist L, Zeeb H, Dragano N, Schmidt B, Ahrens W, Berger K, Castell S, Fricke J, Führer A, Gastell S, Greiser H, Guo F, Jaeschke L, Jochem C, Jöckel K-H, Kaaks R, Koch-Gallenkamp L, Becher H. Persons with migration background in the German National Cohort (NAKO) - sociodemographic characteristics and comparisons with the German autochthonous population. Bundesgesundheitsbl. 2020;63:279–89. 10.1007/s00103-020-03097-9. 10.1007/s00103-020-03097-9 32034443

[37] Statistisches Bundesamt (Destatis). Ausländer in Berlin nach Staatsangehörigkeit| Statista. Ausländer in Berlin Nach Staatsangehörigkeit. 2025. https://de.statista.com/statistik/daten/studie/1094889/umfrage/anzahl-der-auslaender-in-berlin-nach-staatsangehoerigkeit/.

[38] United Nations Educational Scientific and Cultural Organization (UNESCO). International Standard Classification of Education ISCED. 1997.

[39] Dragano N, Bobak M, Wege N, Peasey A, Verde PE, Kubinova R, et al. Neighbourhood socioeconomic status and cardiovascular risk factors: a multilevel analysis of nine cities in the Czech Republic and Germany. BMC Public Health. 2007;7:1–12. 10.1186/1471-2458-7-255. 17888149 10.1186/1471-2458-7-255 PMC2099437

[40] Karriker-Jaffe KJ. Neighborhood socioeconomic status and substance use by U.S. adults. Drug Alcohol Depend. 2013;133(1):212–21. 10.1016/j.drugalcdep.2013.04.033. 23726978 10.1016/j.drugalcdep.2013.04.033 PMC3786055

[41] Mohammed SH, Habtewold TD, Birhanu MM, Sissay TA, Tegegne BS, Abuzerr S, et al. Neighbourhood socioeconomic status and overweight/obesity: a systematic review and meta-analysis of epidemiological studies. BMJ Open. 2019;9(11):1–12. 10.1136/bmjopen-2018-028238. 10.1136/bmjopen-2018-028238 PMC6886990 31727643

[42] Fan JX, Wen M, Li K. Associations between obesity and neighborhood socioeconomic status: variations by gender and family income status. SSM Popul Health. 2020;10:100529. 10.1016/j.ssmph.2019.100529. 31890849 10.1016/j.ssmph.2019.100529 PMC6928347

[43] Schneider S, Gruber J. Neighbourhood deprivation and outlet density for tobacco, alcohol and fast food: first hints of obesogenic and addictive environments in Germany. Public Health Nutr. 2013;16(7):1168–77. 10.1017/S1368980012003321. 22781559 10.1017/S1368980012003321 PMC10271855

[44] van Diepen RJ, van Erpecum CPL, Tabak D, van Zon SKR, Bültmann U, Smidt N. Neighborhood socioeconomic differences in BMI: the role of fast-food outlets and physical activity facilities. Obesity. 2023;31(2):506–14. 10.1002/oby.23617. 36575140 10.1002/oby.23617 PMC10107820

[45] Fox KR, Hillsdon M, Sharp D, Cooper AR, Coulson JC, Davis M, et al. Neighbourhood deprivation and physical activity in UK older adults. Health Place. 2011;17(2):633–40. 10.1016/j.healthplace.2011.01.002. 21292536 10.1016/j.healthplace.2011.01.002

[46] Boone-heinonen J, Diez AV, Kiefe CI, Lewis CE, Guilkey DK, Gordon-larsen P. Social science & medicine neighborhood socioeconomic status predictors of physical activity through young to middle adulthood: the CARDIA study. Soc Sci Med. 2011;72(5):641–9. 10.1016/j.socscimed.2010.12.013. 21316829 10.1016/j.socscimed.2010.12.013 PMC3061839

[47] Deutsche Hauptstelle für Suchtfragen e.V. Alkohol am Arbeitsplatz. In DHS Factsheet. 2019. 10.1024/0040-5930.57.4.270.

[48] Ortiz C, López-Cuadrado T, Rodríguez-Blázquez C, Simón L, Perez-Vicente R., Merlo J, et al. Physical and social environmental factors related to co-occurrence of unhealthy lifestyle behaviors. Health Place. 2022;75. 10.1016/j.healthplace.2022.102804. 10.1016/j.healthplace.2022.102804 35462183

[49] 01.1 Reale Nutzung - Berlin.de. (n.d.). Retrieved December 26, 2025, from https://www.berlin.de/umweltatlas/nutzung/flaechennutzung/2015/karten/artikel.1008651.php.

