# Supplementary material for: Is neighborhood socioeconomic status associated with health behavior in Berlin? Cross-sectional data of the German National Cohort (NAKO)
Source: BMC Public Health. 2026 Feb 23;26:1049. doi: 10.1186/s12889-026-26734-5 (PMC13037057; doi:10.1186/s12889-026-26734-5)
Supplement: Supplementary file 1 — Supplementary Material 1. Supplementary Table 1. Calculation of Healthy Lifestyle Index (HLI). Supplementary Table 2. Characteristics of the study sample stratified by neighborhood SES and the total sample (n = 24,936) including also participants without accelerometry. Supplementary Table 3. Multivariable linear (outcome: Healthy Lifestyle Index HLI) and logistic (outcomes: normal weight, upper median of physical activity (PA), never smoking, no risky alcohol consumption vs respective counterparts) univariate and multivariable regression analyses; multivariable analyses: N = 10,827; adjusted for sex, age, Turkish descent, and education. Supplementary Table 4. Multivariable linear (outcome: Healthy Lifestyle Index HLI) and logistic (outcomes: normal weight, upper median of physical activity (PA), never smoking, no risky alcohol consumption vs respective counterparts) regression analyses; multivariable analyses: N=10,827; adjusted for sex, age, Turkish descent, and education. Supplementary Table 5. Sex-stratified multivariable linear (outcome: Healthy Lifestyle Index HLI) and logistic (outcomes: normal weight, upper median of physical activity (PA), never smoking, no risky alcohol consumption vs respective counterparts) regression analyses; multivariable analyses: N = 10,827; adjusted for sex, age, Turkish descent, and education. Supplementary Figure 1. Association of the Mean Healthy Lifestyle Index (± 1 standard deviation) with nSES. Supplementary Figure 2. Conceptual framework of nSES, covariates and potential environmental confounders/mediators with Healthy Lifestyle Index. [file 12889_2026_26734_MOESM1_ESM.docx]

**Supplementary Table 1.** Calculation of Healthy Lifestyle Index (HLI).

| Modifiable Lifestyle Factors | Lifestyle categories | Category definition | Points  (0=unhealthiest, 3=healthiest) | Dichotomised HLI for regression analysis (1=healthy, 0=unhealthy) |
| --- | --- | --- | --- | --- |
| Body Mass Index (BMI) | Normal weight | <25 kg/m^2^ (normal weight) | 3 | 1 |
|  | Overweight | 25-<30 kg/m^2^ | 2 | 0 |
|  | Obesity (class 1) | 30-<35 kg/m^2^ | 1 |  |
|  | Obesity (class 2 or higher) | ≥35 kg/m^2^ | 0 |  |
| Accelerometer-measured moderate-to-vigorous physical activity in min/day | High | 4th quartile (>88.9-317) | 3 | 1 |
|  | Moderate | 3rd quartile (>65.5-88.9) | 2 |  |
|  | Light | 2nd quartile (>45.4-65.5) | 1 | 0 |
|  | Low | 1st quartile (≤45.5) | 0 |  |
| Smoking Status |  | Never | 3 | 1 |
|  |  | Former | 2 | 0 |
|  |  | Current <15 cigarettes per day | 1 |  |
|  |  | Current ≥15 cigarettes per day | 0 |  |
| Alcohol consumption (AUDIT-C-Score) | Low risk | 0-3 (men)  0-2 (women) | 3 | 1  (<4 (men); <3 (women)) |
|  | Moderate risk | 4-5 (men)  3-5 (women) | 2 | 0  (≥4 (men); ≥3 (woman)) |
|  | High risk | 6-7 | 1 |  |
|  | Severe risk | 8-12 | 0 |  |
| **Total Healthy Lifestyle Index** |  |  | **Max points: 12** |  |

**Supplementary Table 2.** Characteristics of the study sample stratified by neighborhood SES and the total sample (n=24,936) including also participants without accelerometry.

|  |  |  | Neighborhood SES | | | | | | |
| --- | --- | --- | --- | --- | --- | --- | --- | --- | --- |
|  | N | Total | 1 (best) | 2 | 3 | 4 | 5 | 6 | 7 (worst) |
|  |  | 24,936 | 2,100 (8.4) | 4,977 (20.0) | 4,500 (18.0) | 5,514 (22.1) | 3,492 (14.0) | 2,278 (9.1) | 2,075 (8.3) |
| Number of prognosis areas |  | 53 | 8 | 8 | 7 | 7 | 8 | 8 | 7 |
|  |  |  | N (%) or mean±SD | | | | | | |
| Sex | 24,936 |  |  |  |  |  |  |  |  |
| Male |  | 12,282 (49.3) | 1,045 (49.8) | 2,331 (46.8) | 2,291 (50.9) | 2,601 (47.2) | 1,776 (50.9) | 1,180 (51.8) | 1,058 (51.0) |
| Female |  | 12,654 (50.7) | 1,055 (50.2) | 2,646 (53.2) | 2,209 (49.1) | 2,913 (52.8) | 1,716 (49.1) | 1,098 (48.2) | 1,017 (49.0) |
| Age | 24,936 | 49.8±12.9 | 50.7±12.3 | 51.4±12.4 | 48.8±12.5 | 50.4±12.9 | 48.9±12.9 | 49.2±13.8 | 48.4±13.7 |
| Education (ISCED 97)^1^ | 22,357 |  |  |  |  |  |  |  |  |
| High |  | 14,601 (65.3) | 1,340 (70.0) | 3,165 (70.6) | 2,835 (70.4) | 3,152 (63.2) | 1,987 (64.0) | 1,110 (55.0) | 1,012 (55.6) |
| Middle |  | 7,236 (32.4) | 558 (29.2) | 1,269 (28.3) | 1,123 (27.9) | 1,714 (34.3) | 1,029 (33.2) | 827 (41.0) | 716 (39.4) |
| Low |  | 520 (2.3) | 16 (0,8) | 51 (1.1) | 68 (1.7) | 125 (2.5) | 88 (2.8) | 81 (4.0) | 91 (5.0) |
| Employment status | 24,704 |  |  |  |  |  |  |  |  |
| Employed |  | 18,915 (76.6) | 1,618 (77.7) | 3,845 (77.8) | 3,617 (81.2) | 4,109 (75.1) | 2,677 (77.4) | 1,591 (70.5) | 1,458 (71.4) |
| Not employed |  | 978 (4.0) | 42 (2.0) | 150 (3.0) | 150 (3.4) | 213 (3.9) | 138 (4.0) | 149 (6.6) | 136 (6.7) |
| Economically inactive  (retired or other reasons) |  | 4,811 (19.5) | 422 (20.3) | 947 (19.2) | 687 (15.4) | 1,148 (21.0) | 642 (18.6) | 516 (22.9) | 449 (22.0) |
| Monthly net equivalent household income in €^2^ | 23,304 | 2,287±1,491 | 2,701±1,607 | 2,543±1,647 | 2,487±1,666 | 2,165±1,266 | 2,193±1,427 | 1,935±1,286 | 1,704±1,070 |
| Marital status | 24,851 |  |  |  |  |  |  |  |  |
| Living with partner |  | 15,806 (63.6) | 1,562 (74.5) | 3,359 (67.7) | 2,848 (63.5) | 3,382 (61.5) | 2114 (60.8) | 1388 (61.1) | 1,153 (55.8) |
| Not living with partner |  | 2,965 (11.9) | 181 (8.6) | 517 (10.4) | 561 (12.5) | 676 (12.3) | 463 (13.3) | 291 (12.8) | 276 (13.3) |
| No partner |  | 6,080 (24.5) | 353 (16.8) | 1,087 (21.9) | 1,073 (23.9) | 1,437 (26.2) | 898 (25.8) | 593 (26.1) | 639 (30.9) |
| Turkish descent^3^ | 24,921 | 503 (2.0) | 25 (1.2) | 60 (1.2) | 62 (1.4) | 104 (1.9) | 65 (1.9) | 57 (2.5) | 130 (6.3) |
| Lifestyle factors |  |  |  |  |  |  |  |  |  |
| BMI^4^ | 24,179 | 25.9±4.8 | 25.6±4.5 | 25.8±4.7 | 25.5±4.5 | 26.0±4.9 | 25.7±4.7 | 26.7±5.3 | 26.6±5.5 |
| Underweight |  | 352 (1.5) | 25 (1.2) | 73 (1.5) | 59 (1.3) | 79 (1.5) | 57 (1.7) | 31 (1.4) | 28 (1.4) |
| Normal weight |  | 11,373 (47.0) | 1,008 (49.4) | 2,273 (47.6) | 2,172 (49.5) | 2,492 (46.8) | 1,644 (48.3) | 903 (40.7) | 881 (43.6) |
| Overweight |  | 8,377 (34.6) | 717 (35.1) | 1,656 (34.7) | 1,530 (34.9) | 1,861 (34.9) | 1,166 (34.2) | 789 (35.6) | 658 (32.6) |
| Obesity |  | 4,077 (16.9) | 290 (14.2) | 777 (16.3) | 628 (14.3) | 895 (16.8) | 538 (15.8) | 495 (22.3) | 454 (22.5) |
| Physical activity (MVPA-minutes per day)^5^ | 11,922 | 69.8±34.2 | 68.5±34.2 | 70.5±33.7 | 70.9±32.7 | 69.7±34.6 | 70.5±34.5 | 67.3±35.2 | 68.0±35.7 |
| Smoking status | 23,715 |  |  |  |  |  |  |  |  |
| Never |  | 10,370 (43.7) | 1,014 (50.2) | 2,152 (45.6) | 1,932 (44.7) | 2,215 (43.1) | 1,350 (40.2) | 963 (44.1) | 744 (37.9) |
| Former |  | 7,847 (33.1) | 666 (33.0) | 1,649 (34.9) | 1,408 (32.6) | 1,758 (34.2) | 1,105 (32.9) | 646 (29.6) | 615 (31.3) |
| Current |  | 5,451 (23.0) | 336 (16.6) | 916 (19.4) | 977 (22.6) | 1,162 (22.6) | 898 (26.7) | 570 (26.1) | 592 (30.2) |
| Alcohol consumption (AUDIT-C Score)^6^ | 23,612 |  |  |  |  |  |  |  |  |
| Risky alcohol consumption AUDIT-C Score ≥4 (men)/ ≥3 (women) |  | 8,781 (37.2) | 665 (33.0) | 1,789 (38.0) | 1,664 (38.6) | 1,851 (36.1) | 1,351 (40.4) | 739 (34.1) | 722 (37.1) |
| Subjective health status | 23,665 |  |  |  |  |  |  |  |  |
| (Good, very good, excellent) vs. (fair, poor) |  | 15,031 (63.5) | 1,243 (61.5) | 2,970 (62.8) | 2,626 (60.7) | 3.294 (64.3) | 2,094 (62.5) | 1,474 (68.1) | 1,330 (68.3) |
| Life satisfaction  (0=worst, 10=best) | 23,670 | 7.6±1.9 | 7.9±1.7 | 7.7±1.8 | 7.6±1.9 | 7.5±2.0 | 7.6±1.9 | 7.6±2.0 | 7.5±2.0 |

^1^ISCED97: International standard classification of education.(1); ^2^Net household income devided by corresponding household members; ^3^Turkish descent, if i) own Turkish background or ii) migration of at least one parent from Turkey to Germany after 1949 (Wiessner et al., 2020); 4 BMI: Body Mass index in kg/m²; underweight: <18.5kg/m², normal weight: 18.5-24.9 kg/m², overweight: 25.0-29.9 kg/m², obesity: >30.0 kg/m²); ^5^MVPA: moderate-to-vigorous physical activity; 6 AUDIT-C: The AUDIT Alcohol Consumption Questions (2).

1. United Nations Educational Scientific and Cultural Organization (UNESCO). International Standard Classification of Education ISCED. 1997. 1–49 p.

2. Bush K, Kivlahan DR, McDonell MB, Fihn SD, Bradley KA. The AUDIT Alcohol Consumption Questions (AUDIT-C). Arch Intern Med. 1998;158:1789–95.

**Supplementary Table 3.** Multivariable linear (outcome: Healthy Lifestyle Index HLI) and logistic (outcomes: normal weight, upper median of physical activity (PA), never smoking, no risky alcohol consumption vs respective counterparts) univariate and multivariable regression analyses; multivariable analyses: N=10,827; adjusted for sex, age, Turkish descent, and education.

|  | **Healthy Lifestyle Index** | | **Normal weight** | | **Upper median PA** | | **Never smoking** | | **No risky alcohol consumption** | |
| --- | --- | --- | --- | --- | --- | --- | --- | --- | --- | --- |
|  | beta coefficient (univariate) (95% CI) | beta coefficient (adjusted) (95% CI) | Crude OR (95% CI) | Adjusted OR (95% CI) | Crude OR  (95% CI) | Adjusted OR  (95% CI) | Crude OR  (95% CI) | Adjusted OR  (95% CI) | Crude OR  (95% CI) | Adjusted OR  (95% CI) |
| **Social index (per unit worsening)** | -0.08  (-0.10; -0.06) | -0.08  (-0.10; -0.06) | 0.96  (0.94;0.98) | 0.95  (0.93;0.97) | 0.99  (0.97;1.01) | 0.98  (0.96; 1.01) | 0.95  (0.93; 0.97) | 0.96  (0.94; 0.98) | 1.01  (0.99; 1.03) | 1.0  (0.98; 1.03) |
| Sex Ref: Male | 0.32 (0.24;0.39) | 0.33  (0.25; 0.40) | 1.91  (1.78;2.06) | 2.04  (1.89;2.20) | 0.74  (0.69;0.79) | 0.72  (0.67; 0.78) | 1.18  (1.09;1.27) | 1.20  (1.11; 1.30) | 1.3  (1.2;1.4) | 1.31  (1.21; 1.41) |
| Age (per year increase ) | -0.04  (-0.04; -0.04) | -0.04  (-0.04; -0.04) | 0.68  (0.67; 0.68) | 0.96  (0.96;0.96) | 0.96  (0.96;0.97) | 0.96  (0.96; 0.96) | 0.98  (0.98;0.99) | 0.98  (0.98; 0.99) | 1.0  (1.00;1.01) | 1.00  (1.00; 1.01) |
| Turkish descent (Ref: no) | -0.53  (-0.89;-0.17) | -0.34  (-0.69; 0.01) | 0.36  (0.25;0.52) | 0.40  (0.25;0.55) | 0.57  (0.42;0.79) | 0.52  (0.38; 0.73) | 0.69  (0.48;0.97) | 0.23  (0.53; 1.15) | 3.55  (2.19;5.78) | 3.26  (2.00; 5.31) |
| Low education (Ref: high) | -1.25  (-1.53;-0.97) | -1.29  (-1.56; -1.01) | 0.40  (0.31;0.52) | 0.48  (0.36;0.64) | 0.48  (0.37;0.61) | 0.54  (0.42; 0.71) | 0.28  (0.2;0.39) | 0.22  (0.19; 0.41) | 1.87  (1.37;2.55) | 1.65  (1.20; 2.26) |
| Middle education (Ref: high) | -0.51  (-0.6;-0.43) | -0.51  (-0.59; -0.43) | 0.72  (0.66;0.77) | 0.71  (0.66;0.77) | 0.72  (0.67;0.78) | 0.75  (0.69; 0.81) | 0.6  (0.56;0.65) | 0.61  (0.56; 0.66) | 1.21  (1.11;1.32) | 1.19  (1.10; 1.30) |

**Supplementary Table 4.** Multivariable linear (outcome: Healthy Lifestyle Index HLI) and logistic (outcomes: normal weight, upper median of physical activity (PA), never smoking, no risky alcohol consumption vs respective counterparts) regression analyses; multivariable analyses: N=10,827; adjusted for sex, age, Turkish descent, and education.

|  | **Healthy Lifestyle Index** | **Normal weight** | **Upper median PA** | **Never smoking** | **No risky alcohol consumption** |
| --- | --- | --- | --- | --- | --- |
|  | Adjusted* beta coefficient (95% CI) | Adjusted* OR (95% CI) | Adjusted* OR  (95% CI) | Adjusted* OR  (95% CI) | Adjusted* OR  (95% CI) |
| **Social index**  **Highest (1-4)** | Ref. | Ref. | Ref. | Ref. | Ref. |
| **Lowest (5-7)** | -0.3 (-0.38;-0.22) | 0.86 (0.79;0.94) | 0.93 (0.85;1.01) | 0.88 (0.80;0.95) | 0.97 (0.89;1.06) |

*adjusted for sex, age, education, and migrant background

**Supplementary Table 5.** Sex-stratified multivariable linear (outcome: Healthy Lifestyle Index HLI) and logistic (outcomes: normal weight, upper median of physical activity (PA), never smoking, no risky alcohol consumption vs respective counterparts) regression analyses; multivariable analyses: N=10,827; adjusted for sex, age, Turkish descent, and education.

|  | **Healthy Lifestyle Index** | | **Normal weight** | | **Upper median PA** | | **Never smoking** | | **No risky alcohol consumption** | |
| --- | --- | --- | --- | --- | --- | --- | --- | --- | --- | --- |
|  | Men | Women | Men | Women | Men | Women | Men | Women | Men | Women |
|  | Adjusted* beta coefficient (95% CI) | | Adjusted* OR (95% CI) | | | | | | | |
| **Social index**  **Highest (1-4)** | Ref. | | | | | | | | | |
| **Lowest (5-7)** | -0.28 (0.16;0.40) | -0.31 (0.20;0.42) | 0.99 (0.87;1.12) | 0.76  (0.67;0.85) | 0.95 (0.84;1.07) | 0.91 (0.81;1.02) | 0.93 (0.82;1.05) | 0.83 (0.74;0.94) | 0.90 (0.80;1.02) | 1.05 (0.93;1.19) |
| **p(interaction)** | 0.692 | | 0.002 | | 0.481 | | 0.203 | | 0.055 | |

*adjusted for age, education, and migrant background


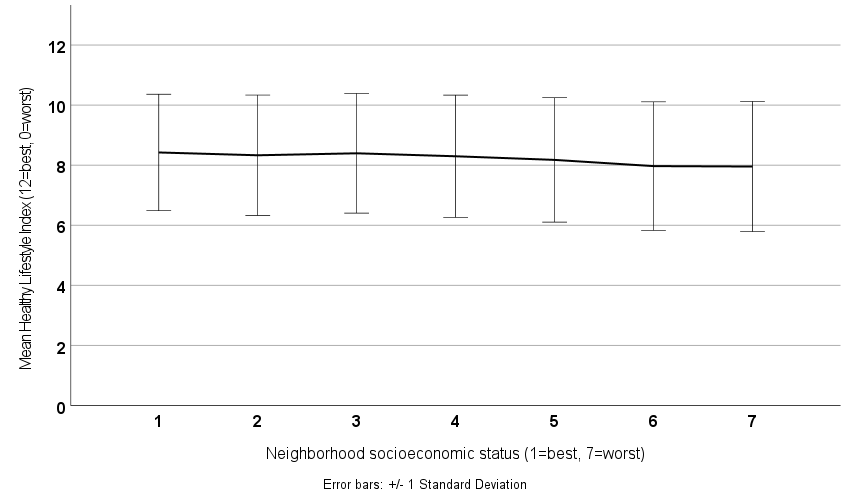


**Supplementary Figure 1.** Association of the Mean Healthy Lifestyle Index (± 1 standard deviation) with nSES.


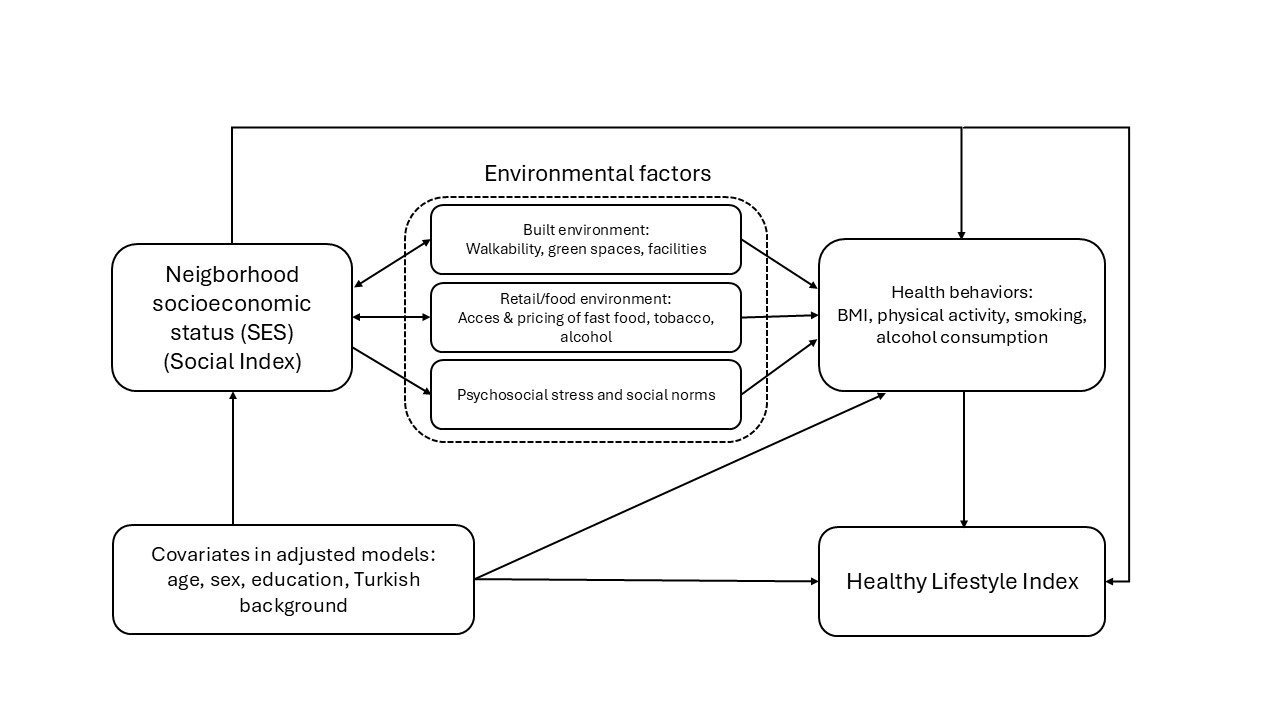


**Supplementary Figure 2.** Conceptual framework of nSES, covariates and potential environmental confounders/mediators with Healthy Lifestyle Index.
